# Primary Causes of Hospitalizations and Procedures, Predictors of In-hospital Mortality, and Trends in Cardiovascular and Cerebrovascular Events Among Recreational Marijuana Users: A Five-year Nationwide Inpatient Assessment in the United States

**DOI:** 10.7759/cureus.3195

**Published:** 2018-08-23

**Authors:** Rupak Desai, Sofia Shamim, Krupa Patel, Ashish Sadolikar, Vikram Preet Kaur, Siddhi Bhivandkar, Smit Patel, Sejal Savani, Zeeshan Mansuri, Zabeen Mahuwala

**Affiliations:** 1 Division of Cardiology, Atlanta Veterans Affairs Medical Center, Decatur, USA; 2 Family Medicine, Dekalb Medical Centre, Atlanta, USA; 3 Medicine, Avalon University School of Medicine, Willemstad, CUW; 4 Department of Internal Medicine and Psychiatry, Florida International University, Miami, USA; 5 Division of Psychiatry, Chicago Lakeshore Hospital, Illinois, USA; 6 Department of Psychiatry, Smolensk State Medical University, Smolensk, RUS; 7 Neurology, Hartford Hospital, Hartford, USA; 8 New York University, New York, USA; 9 Psychiatry, Texas Tech University Health Sciences Center at Odessa, Midland, USA; 10 Neurology, University of Kentucky, Lexington, USA

**Keywords:** marijuana legalization, recreational marijuana, cannabis, comorbidities, procedures, in-hospital mortality, cardiovascular diseases, cerebrovascular disease, complications, length of stay

## Abstract

Background

Recent trends in the legalization of marijuana in many states are increasing the popularity of recreational marijuana use. Since current data on hospitalizations in marijuana users is sparse, we evaluated the primary reasons for admissions, procedures and associated healthcare burden in hospitalized recreational marijuana users.

Methods

The National Inpatient Sample (NIS) for the years 2010–2014 was queried for the hospitalizations with a history of recreational marijuana usage using applicable ICD-9 CM codes. Descriptive statistics were used to report frequency (N) and percentage (%). Discharge weights were applied to achieve national estimates. The predictors of in-hospital mortality in recreational marijuana users were assessed using a two-way hierarchical multivariate regression after adjusting for the confounders.

Results

We analyzed 465,959 (weighted n=2,317,343) hospitalizations with a history of recreational marijuana use. Among psychiatric disorders, most prominent primary discharge diagnoses were mood disorders (20.6%), schizophrenia/other psychotic disorders (10.6%), and substance/alcohol-related disorders (10.4%). Suicide and intentional self-inflicted injury (3.6%) was the leading cause of emergency admission. The most common non-psychiatric primary discharge diagnoses were diabetes mellitus with chronic complications (2.2%), acute myocardial infarction (AMI) (1.2%), nonspecific chest pain (1.1%), congestive cardiac failure (CHF) (1%), arrhythmia (0.8%), and hypertension (0.8%). Acute cerebrovascular diseases were noted in 1.1% and epilepsy in 1.8% of patients. Alcohol/drug rehabilitation and detoxification (6.9%) and psychiatric evaluation/therapy (3.9%) were the most evident psychiatric procedures whereas most frequent non-psychiatric procedures were diagnostic coronary arteriography (1%), percutaneous transluminal coronary angioplasty (0.7%), and echocardiogram (0.7%). Top independent predictors of in-hospital mortality were coagulopathy (OR 5.94), AMI (OR 4.59), pulmonary circulation disorder (OR 2.95), CHF (OR 2.02), renal failure (OR 1.91), coronary atherosclerosis (OR 1.34) and peripheral vascular disorder (OR 1.31). Major cardiovascular and cerebrovascular events also showed increasing trends among users.

Conclusion

We established the most frequent psychiatric and non-psychiatric causes of admissions and procedures in recreational marijuana users, which may pose a significant healthcare burden and increase the odds of in-hospital mortality.

## Introduction

Many states in the United States (US) have recently shown tremendous progress in making amendments favoring the legalization of medicinal and recreational marijuana [1]. Recent trends in marijuana usage have confirmed the growing popularity of recreational marijuana use especially among youth in the United States (US) [2]. In light of the increase in trends of legalization and decriminalization of marijuana and marijuana use among youth, an intersection between marijuana use and the existing disease burden in America is plausible but currently understudied. As more states approve recreational use, there is a reintroduced urgency to learn about its effects and causes of unpredictable hospital admissions and procedures in marijuana users. The aim of this study is to assess the most frequently identified causes of hospitalization, procedures, trends in cardiovascular and cerebrovascular events, and independent predictors of in-hospital mortality in recreational marijuana users from 2010 through 2014 using the largest nationwide cohort.

## Materials and methods

Data source

We queried the largest inpatient database in the US, the National Inpatient Sample (NIS), for the years 2010 to 2014. The NIS, part of the Healthcare Cost and Utilization Project (HCUP) sponsored by Healthcare Research and Quality Agency (AHRQ), is publically available and estimates 20% sample admissions from 1000 US hospitals in more than 40 states. It contains average 7-8 million unweighted discharges each year. Discharge weights provided by HCUP were applied to calculate national estimates; estimating more than 35 million weighted hospitalizations in the US population. The NIS provides one primary discharge diagnosis and up to twenty-four secondary discharge diagnoses, and one primary procedure with up to fourteen secondary procedures data. The data on diagnoses and procedures in the NIS are identified using the International Classification of Diseases, Ninth Edition, Clinical Modification (ICD-9 CM). More information on HCUP-NIS is available at https://www.hcup-us.ahrq.gov/nisoverview.jsp.

Study population

With a specific end goal to assess the links between marijuana use and clinical results among hospitalized patients in the NIS database between 2010 and 2014, recreational marijuana users were identified based on ICD-9 CM codes 304.30, 304.31, 304.32, 305.20, 305.21, and 305.22. This approach was used in an earlier study by Desai et al. to identify recreational marijuana users in the NIS database [3]. Since there is no precise ICD-9-CM code for medicinal cannabis use, we made the assumption that patients with clinical findings of cannabis use were likely to consume recreational marijuana. Several studies show that a large share of medicinal users does, in fact, use cannabis recreationally [4-5]. In addition, a recent investigation assessed that roughly 86% of individuals who report regular use of cannabis for therapeutic purposes also utilize it for recreational purposes [6]. Hence, the ICD-9-CM codes for cannabis abuse were utilized for recreational marijuana use in this study.

Statistical analyses

Descriptive statistics and Student's t-test were used to assess the baseline demographics and hospital characteristics for patients hospitalized with recreational marijuana history. A two-tailed p-value of <0.05 was considered to determine the statistical significance. The categorical variables and continuous variables were expressed in percentages and Mean ± SD format respectively. We evaluated the odds of in-hospital mortality in hospitalized recreational marijuana users by univariate analysis. Subsequently, clinically significant patients’ variables were integrated into multivariate logistic regression. Multivariate analysis results were reported in terms of the adjusted odds ratio, 95% confidence interval, and p-value. The multivariate regression model was adjusted for demographics, hospital characteristics, relevant comorbid conditions, and other drug abuse to evaluate the odds of in-hospital mortality among recreational marijuana users. IBM SPSS Statistics 22.0 (IBM Corp, Armonk, New York) software was used to execute all statistical analyses. Since the NIS database does not disclose patients' identification details, the study was exempt from the Institutional Review Board approval.

Outcome measures

In-hospital mortality was defined by all-cause deaths occurred during hospitalization. Length of stay was measured in days (Mean±SD) and total hospital charges were expressed in mean USD. All baseline patient and hospital characteristics and primary causes of admission and procedures in recreational marijuana users are expressed in frequency (N) with percentages (%). Trends in cardiovascular and cerebrovascular disease-related hospitalizations were assessed using the linear-by-linear association.

## Results

Baseline characteristics of the study population

Our analysis included 465,959 (weighted N=2,317,343) hospitalizations with a history of recreational marijuana use in the US between 2010 and 2014. The study population consisted of a majority of patients in the 18–44 years age range with the mean as 37 years. The highly represented racial groups were White (54.0%), African American (31.6%), and Hispanic (9.3%) and almost two-thirds of patients were males (63.2%). Most hospital admissions were non-elective (87.3%). Recreational marijuana-related admissions were predominantly noted in the South region (33.2%) and in urban hospitals (91.2%). Within urban areas, more admissions were documented at teaching hospitals (58.9%) compared to non-teaching hospitals (32.3%). Most of the study population comprised of Medicaid/Medicare enrollees. The mean duration of hospitalization was 5 days, with an average of $28,916 in total hospital care charges. All-cause in-hospital mortality rate was 0.5% among recreational marijuana users (Table 1).

**Table 1:** Baseline Demographics and Hospital Characteristics of the Study Population (N=465,959, weighted N=2,317,343) HMO= health maintenance organization, SNF= skilled nursing facility, ICF=intermediate care facility The bed size cutoff points distributed into small, medium, and large. Derived from https://www.hcup-us.ahrq.gov/db/vars/hosp_bedsize/nisnote.jsp ^#^ The quartiles are identiﬁed by values of 1 to 4, indicating the poorest to wealthiest populations. Derived from https://www.hcup-us.ahrq.gov/db/vars/zipinc_qrtl/nisnote.jsp

Variables	Frequency (N)	Percent (%)
Age in years at hospitalization		
Mean age (Mean±SD)	37±13	
18-44	1,590,826	68.6%
45-64	673,848	29.1%
65-84	52,002	2.2%
≥85	667	0.03%
Indicator of Sex		
Male	1,463,657	63.2%
Female	853,174	36.8%
Admission type		
Non-elective	2,015,030	87.3%
Elective	293,952	12.7%
Admission day		
Weekday (Mon-Fri)	1,772,528	76.5%
Weekend (Sat-Sun)	544,811	23.5%
Race		
White	1,155,490	54.0%
African American	675,172	31.6%
Hispanic	199,737	9.3%
Asian and Pacific Islander	17,410	0.8%
Native American	22,654	1.1%
Others	68,343	3.2%
Disposition		
Routine	1,893,569	81.8%
Transfer to short-term hospital	42,961	1.9%
Other transfers (SNF, ICF, other)	167,033	7.2%
Home Health Care	82,708	3.6%
Against Medical Advice	115,368	5.0%
Median household income percentile for patient's zip code^#^		
0-25^th^	924,222	41.8%
26-50^th^	562,216	25.4%
51-75^th^	436,287	19.7%
76-100^th^	289,999	13.1%
Primary expected payer		
Medicare	377,239	16.3%
Medicaid	871,589	37.7%
Private including HMO	471,935	20.4%
Self – Pay/No charge/Other	588,631	25.5%
Control/ownership of hospital		
Government, nonfederal	373,535	16.2%
Private, non-profit	1,663,245	72.3%
Private, invest-own	264,452	11.5%
Bed size of hospital		
Small	290,988	12.6%
Medium	594,048	25.8%
Large	1,416,195	61.5%
Location/teaching status		
Rural	202,406	8.8%
Urban non-teaching	742,326	32.3%
Urban teaching	1,356,500	58.9%
Region of hospital		
Northeast	497,658	21.5%
Midwest	604,968	26.1%
South	769,810	33.2%
West	444,907	19.2%
Length of stay (days) (Mean±SD)	5±7	
Total hospital charges (Mean)	$28,916	
In-hospital mortality	12360	0.5%

Most frequent causes of hospitalization and procedures performed: psychiatric and non-psychiatric**

Psychiatric

Among psychiatric conditions, the most prominent primary discharge diagnoses were mood disorders (20.6%), schizophrenia and other psychotic disorders (10.6%), substance, and alcohol-related disorders (10.4%) in recreational marijuana users (Table 2). Suicide and intentional self-inflicted injury (3.6%) was the leading cause of emergency admission. Other reasons included poisoning by psychotropic agents, miscellaneous mental health disorders, and adjustment disorders. Noticeable primary procedures were alcohol and drug rehabilitation/detoxification (6.9%) and psychiatric evaluation and therapy (3.9%) (Table 3).

**Table 2:** Most Common Primary Causes of Hospitalization in Recreational Marijuana Users

Disorder	Frequency	%
Mood disorder	477,670	20.6
Schizophrenia and other psychotic disorders	246,444	10.6
Substance-related disorders	137,114	5.9
Alcohol-related disorders	104,500	4.5
Diabetes mellitus with complications	50,510	2.2
Poisoning by other medications and drugs	43,959	1.9
Epilepsy; convulsions	41,972	1.8
Skin and subcutaneous tissue infections	38,453	1.7
Poisoning by psychotropic agents	37,606	1.6
Septicemia (except in labor)	36,800	1.6
Pancreatic disorders (not diabetes)	34,864	1.5
Other complications of pregnancy	26,918	1.2
Acute myocardial infarction	26,664	1.2
Nonspecific chest pain	26,442	1.1
Miscellaneous mental health disorders	26,091	1.1
Acute cerebrovascular disease	25,777	1.1
Adjustment disorders	24,043	1.0
Pneumonia	23,805	1.0
Congestive heart failure; nonhypertensive	23,037	1.0
Asthma	20,637	0.9
Fracture of lower limb	19,512	0.8
Acute and unspecified renal failure	19,470	0.8
Other complications of birth; puerperium	19,465	0.8
Cardiac dysrhythmias	19,321	0.8
Intracranial injury	19,037	0.8
Hypertension	18,391	0.8
Respiratory failure; insufficiency; arrest (adult)	17,680	0.8
Crushing injury or internal injury	17,145	0.7
Chronic obstructive pulmonary disease and bronchiectasis	17,044	0.7
Other disorders of stomach and duodenum	16,355	0.7
Gastrointestinal hemorrhage	15,463	0.7

**Table 3:** Most Frequently Documented Primary Procedures in Recreational Marijuana Users

Procedures	Frequency	%
Alcohol and drug rehabilitation/detoxification	160,721	6.9
Psychological and psychiatric evaluation and therapy	89,640	3.9
Respiratory intubation and mechanical ventilation	62,291	2.7
Other procedures to assist delivery	58,886	2.5
Upper gastrointestinal endoscopy; biopsy	42,657	1.8
Cesarean section	36,700	1.6
Other vascular catheterization (not heart)	27,500	1.2
Other therapeutic procedures	27,494	1.2
Diagnostic cardiac catheterization; coronary arteriography	23,308	1.0
Blood transfusion	18,908	0.8
Percutaneous transluminal coronary angioplasty	16,672	0.7
Incision and drainage; skin and subcutaneous tissue	16,490	0.7
Diagnostic spinal tap	16,236	0.7
Diagnostic ultrasound of heart (echocardiogram)	15,869	0.7
Hemodialysis	15,820	0.7
Repair of current obstetric laceration	14,601	0.6
Prophylactic vaccinations and inoculations	12,845	0.6
Treatment; fracture or dislocation of lower extremity	12,824	0.6
Suture of skin and subcutaneous tissue	12,746	0.6
Other diagnostic procedures	11,326	0.5
Cholecystectomy and common duct exploration	10,174	0.4
Incision of pleura; thoracentesis; chest drainage	9,607	0.4

Non-psychiatric

The non-psychiatric causes of hospitalizations among marijuana users included endocrine, infectious, respiratory, cerebrovascular and circulatory etiologies. Among non-psychiatric reasons, the most common primary discharge diagnoses were acute myocardial infarction (1.2%), nonspecific chest pain (1.1%), congestive cardiac failure (1%), arrhythmia (0.8%), and hypertension (0.8%). Cerebrovascular diseases (stroke) were present in 1.1% and epilepsy (convulsions) in 1.8% of cases. Diabetes mellitus with chronic complications was present in 2.2% of cases. Commonly performed procedures among marijuana users admitted with cardiac causes were diagnostic coronary arteriography (1%), percutaneous transluminal coronary angioplasty (0.7%) and echocardiogram (0.7%). Skin and soft-tissue infections (1.7%), pneumonia (1%) and pancreatic disorders (1.5%) were the most frequent infectious, respiratory and gastrointestinal etiologies for hospitalization respectively (Table 1).

Multivariate predictors of in-hospital mortality

Demographic factors associated with increased odds of in-hospital mortality were male sex (OR 1.18, 95% CI 1.13–1.23) and increasing age (OR 1.05, 95% CI 1.04–1.05). Hospital characteristics associated with an increased odds of in-hospital mortality were admission at an urban teaching hospital (compared to a rural hospital) (OR 1.49, 95% CI 1.38–1.62) and hospitalization in 2014 (compared to 2010) (OR 1.08, 95% CI 1.01–1.15). Non-elective hospital admissions (OR 2.27, 95% CI 2.08–2.48) were associated with increased odds of in-hospital mortality. All p<0.05 were considered clinically significant and denoted graphically in a forest plot.

**Figure 1:**
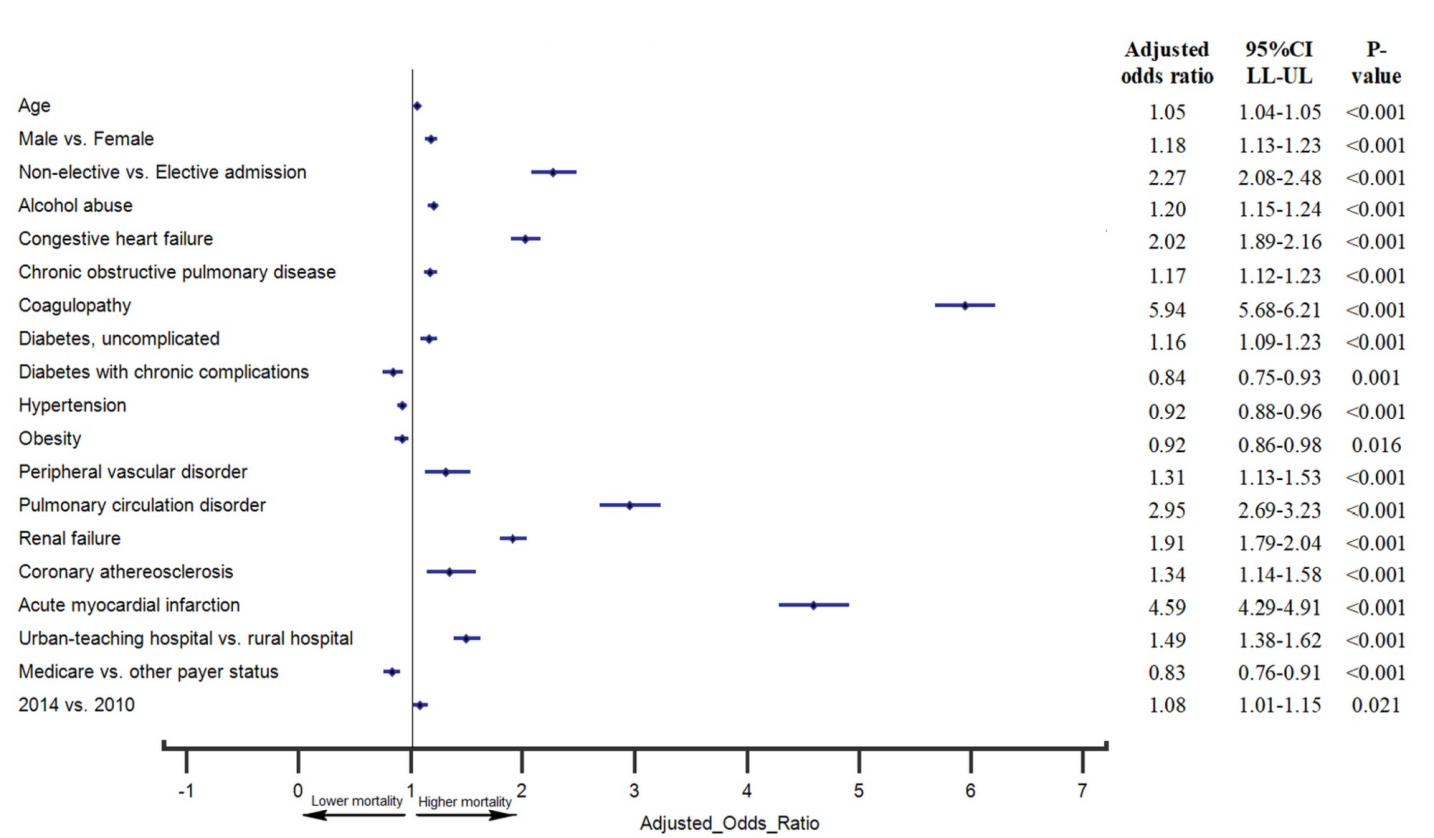
Multivariate Independent Predictors of In-hospital Mortality Among Hospitalized Recreational Marijuana Users Advanced age, male sex, non-elective and urban teaching hospital admissions were found to be independent predictors of higher in-hospital mortality. Comorbidities associated with higher inpatient mortality were coagulopathy, acute myocardial infarction, pulmonary circulation disorder, congestive heart failure, alcohol abuse, chronic obstructive pulmonary disease, diabetes without complications, peripheral vascular disorder, renal failure, and coronary atherosclerosis.

After adjusting for potential confounders, the multivariate analysis revealed that coagulopathy had the strongest association with in-hospital mortality (OR 5.94, 95% CI 5.68-6.21) among hospitalized recreational marijuana users (Figure 1). Other factors independently associated with an increased odds of in-hospital mortality were acute myocardial infarction (OR 4.59, 95% CI 4.29–4.91), pulmonary circulation disorder (OR 2.95, 95% CI 2.69–3.23), congestive heart failure (OR 2.02, 95% CI 1.89–2.16), alcohol abuse (OR 1.20, 95% CI 1.15–1.24), chronic obstructive pulmonary disease (OR 1.17, 95% CI 1.12–1.23), diabetes without complications (OR 1.16, 95% CI 1.09 -1.23), peripheral vascular disorder (OR 1.31, 95% CI 1.13–1.53), renal failure (OR 1.91, 95% CI 1.79–2.04) and coronary atherosclerosis (OR 1.34, 95% CI 1.14–1.58). All P < 0.05 were considered clinically significant and denoted graphically in a forest plot.

Trends in major cardiovascular and cerebrovascular events in recreational marijuana users

**Figure 2:**
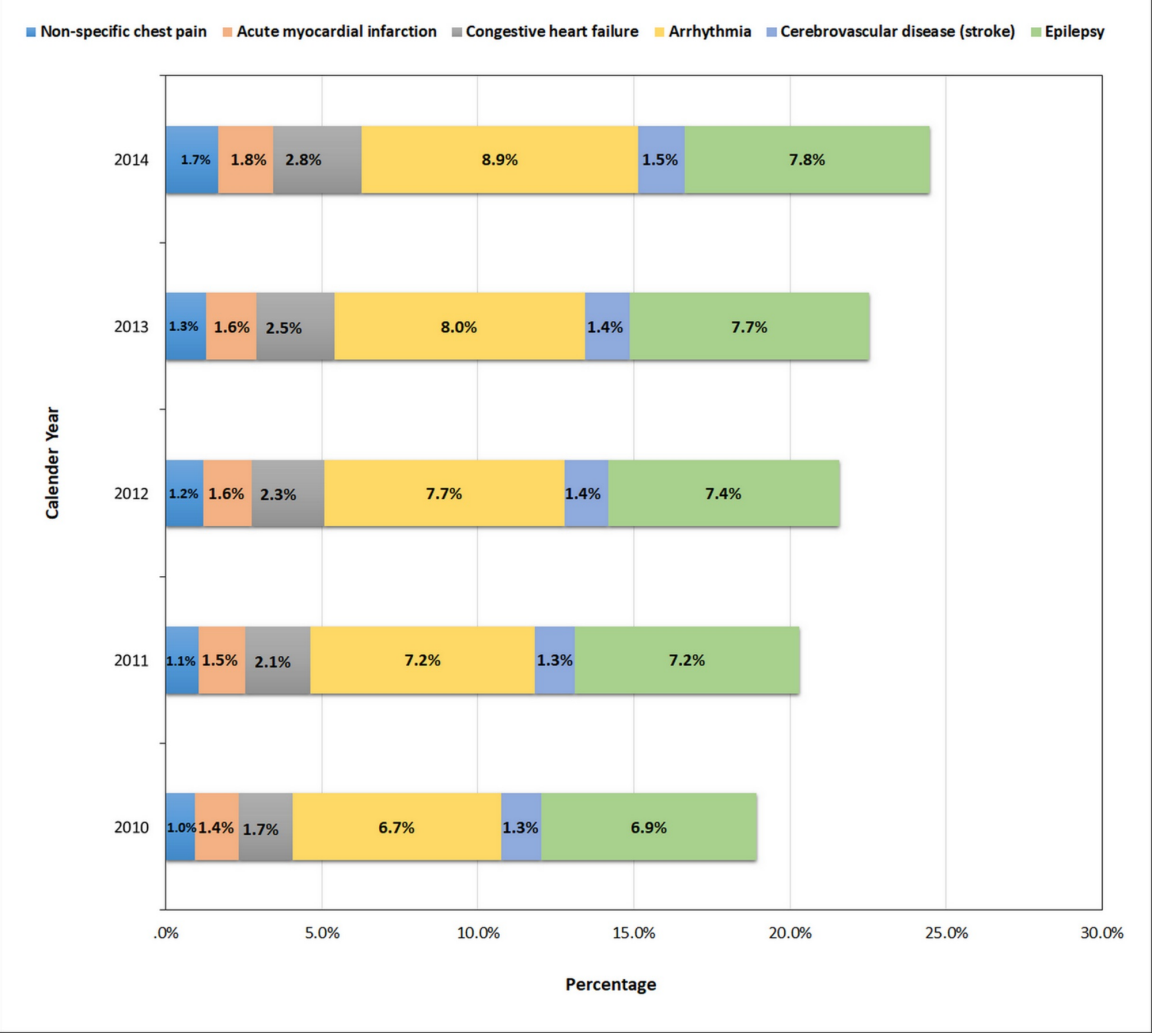
Trends in Cardiovascular and Cerebrovascular Events in Hospitalized Recreational Marijuana Users

As shown in Figure 2, major cardiovascular (non-specific chest pain, acute myocardial infarction, congestive heart failure, arrhythmia) and cerebrovascular (stroke and epilepsy) events showed increasing trends among recreational marijuana users during the study period (p-trend<0.001).

## Discussion

This cross-sectional study helped to establish different primary causes for hospitalization, procedures, trends in cardiovascular-cerebrovascular events, and predictors of in-hospital mortality in marijuana users. Causes of hospitalization were attributed to direct effects of marijuana use while indirect effects were attributed to marijuana use superimposed on pre-existing comorbidities. Most of the hospital admissions in marijuana users were non-elective and had a routine discharge. The higher rate of hospital admissions was noted on weekdays as opposed to the weekends. Demographic analysis of our study showed an increase in the prevalence of recreational marijuana use from 2010 to 2014 for both genders, however, increases were greater for men leading to the widening gap of marijuana use in gender over time.

Marijuana use is believed to be highly connected with unemployment and various hypothesis have been considered ranging from lack of motivation to keep up with the professional competitive environment or mild to moderate cognitive impairment leading to job problems. Corresponding to prior studies, low socioeconomic status was associated with increased use of marijuana in our study as well [7]. The racial distribution showed that whites represented over half of the recreational marijuana users in our study. African Americans were second most frequent consumers of marijuana, whereas, Asians and Pacific Islanders recorded the lowest frequency of marijuana use. Existing studies show that the use of marijuana among the race in the USA differed to converge in 2017 with an increasing number of African American students using the drug at least once or twice during the period of the study [8].

Psychiatric disorders and procedures

Environmental factors, such as drug use, can alter the maturational process of the developing brain in adolescence leading to an increase in the incidence of psychiatric illness and substance abuse. Studies have suggested a toxic effect of long-term cannabis use on white matter development leading to an increased risk of psychosis during adolescence [9]. Use of marijuana can directly affect the brain and influence school performance and engagement in risk/reward behaviors [10]. Unfortunately, United Nation Drug Control Programme (UNDCP 1998) reports that sixty percent of students and youths abuse drugs, which raises significant health concerns [1]. In a study conducted among the students in 2016, shows that 39% of full-time college students aged 19-22 indicated that they used marijuana at least once in the prior 12 months and 22% indicated that they used it at least once in past 30 days [3]. The damages caused by the use of marijuana on the brain can last for a longer time or even be permanent depending on the levels of tetrahydrocannabinol (THC) in marijuana, which is consistent with our study where psychiatric and psychological evaluation and therapy was the most frequently documented primary procedures.

Mood Disorders

Our study substantiated findings of other studies in which the most frequent primary cause of in-hospital admission among recreational marijuana users was mood disorder [11]. Patients who are recreational marijuana users are at higher risk of elevated mood and worse global functioning, prolonged duration of each manic episode, and increased suicidal ideations [12]. National Epidemiologic Survey on Alcohol and Related Conditions by Level of Cannabis Use (2001–2002, 2004–2005) also showed that marijuana users most often presented with mood disorders and anxiety disorders [13].

Schizophrenia and Other Psychotic Disorders

Nearly 11% of recreational marijuana users experienced schizophrenia in our analysis. Previous prospective studies in the general population have shown that cannabis use is associated with a two-fold increase in the risk of psychotic disorders, particularly schizophrenia; a higher risk of schizophrenia is predicted by an earlier onset of cannabis use [14]. A study reports that schizophrenia patients with a history of marijuana use had a significantly higher burden of lifetime in-patient care than non-marijuana users [15]. The appreciable proportion of marijuana users present with psychotic states following heavy consumption and regular users are at risk of dependence. People with major mental illnesses such as schizophrenia are especially vulnerable to marijuana intake, which generally provokes relapse and aggravates existing symptoms [16].

Substance Abuse

Our study reports that eleven percent of marijuana users reported to the hospital with other substance abuse-related disorders and alcohol-related disorders being the third most frequent cause of marijuana-related hospital admissions. Sharma et al. concluded that patients primarily admitted for an alcohol abuse disorders were found to be regular consumers of other substances like marijuana (8%), cocaine (12%), methamphetamine (8%), and tranquilizers (12%) [17].

Drug Toxicity

Poisoning by psychotropic agents and by other drugs was the culprit in 3.5% marijuana-related hospital admissions. The most frequent symptoms observed in acute toxicity of ingested synthetic marijuana-laced brownies have been dry mouth, tingling sensation, memory impairment, lightheadedness, difficulty focusing, blurred vision, and inappropriate laughter [18]. Synthetic cannabinoids, commonly adulterated with other psychoactive drugs, can lead to toxicity with amplified morbidity and mortality.

Non-psychiatric disorders and procedures

Nervous Disorders

Stroke is a leading healthcare problem in the US and a direct and indirect burden of around $68.9 billion was imposed on the US healthcare owing to strokes in 2009. The higher financial burden was owing to illicit drugs use, among which, cocaine and marijuana-related worse outcomes in stroke patients have been reported earlier [19]. THC is thought to mediate its psychotropic effects through the CB1 receptor located in the brain. Epilepsy and convulsions were found to be the most common primary presentations at the time of marijuana-related admission. We observed the trends in stroke prevalence among marijuana users nearly doubled from 2010 to 2014. The self-resolved transient ischemic attacks in patients during marijuana use suggest a reversible effect of marijuana inhalation on the blood vessels of the brain that may be attributable to a spasm [20].

Cardiac Disorders

Cardiovascular disease is the most common chronic illness in both developed and developing countries, causing the most deaths and the greatest impact on morbidity [21]. Cannabis can cause adverse effects on the cardiovascular system by increasing dose-dependent heart rate, which is particularly concerning in pre-existing heart ailments. THC acts primarily on the endocannabinoid system (cannabinoid receptors CB1 and CB2, which are distributed in the central nervous system, cardiovascular system, and peripheral tissues), which regulates cardiovascular function and exerts sympathetic stimulation. Delineated cardiovascular effects of THC are increased heart rate, increased supine blood pressure, orthostatic hypotension, increased cardiac output, reductions in left ventricular ejection time, and increases in venous carboxyhemoglobin levels, which may produce morbid cardiovascular and cerebrovascular outcomes [22].

Over the past decade, there has been a steadily increasing number of case reports describing acute coronary events occurring shortly after marijuana use. Many case reports depict young male patients without any co-morbidity presenting with chest pain, ST elevation, and positive troponin after using marijuana. Several such case reports on toxicology screen reveal positive test results for marijuana only but negative for cocaine, opiates, amphetamines [23], which proves that marijuana can alone be responsible for acute myocardial infarction. Our study also indicated a clear syndication between the two. Acute myocardial infarction showed higher odds of in-hospital mortality when compared with any other pathology among marijuana users. Previous studies show up to 4.8-fold increase in the incidence of myocardial infarction over baseline in the first hour after marijuana use along with 4.6-fold higher odds of mortality owing to acute myocardial infarction [24]. In our study, the frequently performed cardiac interventional procedures among marijuana users are diagnostic cardiac catheterization, coronary arteriography (1.0%), percutaneous transluminal coronary angioplasty (PTCA) (0.9%) and diagnostic ultrasound of the heart (echocardiogram) (0.7%). The low rates of treatment seeking warrant attention in treatment and prevention strategies. A sizable proportion of the cardiovascular effects of marijuana are mediated through activation of the sympathetic nervous system and inhibition of the parasympathetic autonomic nervous system. Heart rate variability, a marker for autonomic dysfunction, can ultimately lead to sudden death from arrhythmia. In our study, we learned that trends in hospital presentation for epilepsy and arrhythmia among the marijuana users increased in recent years consistent with recently published study depicting increasing trends in arrhythmia including all subtypes of arrhythmia in marijuana users [25]. The burden of arrhythmias in epilepsy patients has been well depicted in the previous reports, which can present as parallel occurrences in marijuana users [26].

Vascular Diseases

Our study revealed that coagulopathy had significantly higher odds of in-hospital mortality. Use of marijuana can cause thromboangiitis obliterans (TAO) or cannabis-associated arteritis (CAA). Cannabis arteritis (CA) is a very rare peripheral vascular disease similar to Buerger's disease. It presents as a lower limb peripheral necrosis with the absence of other atherosclerotic risk factors in men aged less than fifty. Cannabis causes potent angiospasms, which could be contributing to the pathogenesis of the disease. The reports suggest that THC affects the platelet function leading to increased aggregation. THC activates platelets by endocannabinoids: 2-arachidonoylglycerol (2-AG) and anandamide and enhances glycoprotein IIb-IIIa and P-selectin expression causing increased platelet aggregation [27]. These pathophysiological effects of marijuana have the potential of causing cerebral hemorrhages and other bleeding disorders.

Systemic Effects

Marijuana is generally an immunosuppressant and hence, overuse can influence the immune system adversely. In the presence of THC, natural killer (NK) cells show diminished production of tumor necrosis factor alpha (TNF-α) when challenged with infection [28]. Correspondingly, we found out that 1.6 % of our study population was primarily admitted with septicemia and 1.7% patients reported skin and subcutaneous tissue infections. The respiratory system can also be adversely affected in marijuana users. Pneumonia, asthma, chronic obstructive pulmonary disease, and bronchiectasis were the most frequent respiratory causes of hospital admissions among marijuana users. This can be attributed to the smoke content of cannabis, which is similar to that of tobacco. Diabetes mellitus and other pancreatic disorders were noted as prominent gastrointestinal etiologies for hospital admissions. Evidence from both preclinical and human studies has indicated that overactivity of the CB1R system contributes to the development of insulin resistance and both type 1 diabetes and type 2 diabetes [29]. The study results showed gastrointestinal hemorrhage as one of the common gastric etiologies of hospitalizations among marijuana users. Since marijuana and gastrointestinal hemorrhage-related readmissions pose a major burden on US healthcare, future preventive strides may help to restrain health care cost in such patients [30].

Predictors of in-hospital mortality in marijuana users

Although acute toxicity is low, the chronic use of marijuana can cause grave complications. Recent advancements in the legalization of marijuana necessitate the determination of mortality associations in marijuana users. Congestive heart failure, coronary atherosclerosis, acute myocardial infarction, peripheral vascular disorder, and coagulopathy were most frequent predictors of the cardiovascular mortality in marijuana users. Other mortality causes are chronic obstructive pulmonary disease, a pulmonary circulation disorder, renal failure and alcohol abuse. These presentations are most often linked to higher mortality in the US. Although we are unable to establish a direct causal association between the mortality and marijuana use, we report the significant portion of the marijuana users demonstrated higher odds. There is a substantial disease burden that affects quality and length of life among marijuana users. Our study can help to cultivate measures for public health and to establish interventions for prevention and to compare the premature mortality experience between populations.

Although findings from the NIS can be generalized to a larger US population, it also bears a few potential limitations. Since it is an administrative dataset, coding errors cannot be denied leading to under or overreporting of the diseases and procedures. The direct causation role cannot be established considering the retrospective nature of the data. We have included only primary discharge diagnoses and procedures which may have underestimated the actual disease burden in some cases. As with any retrospective study, we could not control all the confounders in the trend analysis. With a few limitations, the study nevertheless provides a better insight into the contemporary nationwide estimates.

## Conclusions

In this largest nationwide cross-sectional study, we identified the most frequent causes of hospitalizations and procedures, trends in major cardiovascular and cerebrovascular diseases, and predictors of in-hospital mortality in recreational marijuana users. A substantial disease burden owing to cardiovascular and cerebrovascular events and procedures in addition to expected psychiatric illnesses affect the quality of life among marijuana users and increases the odds of the in-hospital mortality. The mindfulness of most frequently encountered coexisting illness or complications among recreational marijuana users may help clinicians to be extra attentive and screen them during history-taking and follow-up care after discharge. Our study findings can be helpful to cultivate public health measures for prevention of marijuana-related complications and mortality and restrain the healthcare burden posed by marijuana-related hospitalizations.
